# Serum proteome of dogs with chronic enteropathy

**DOI:** 10.1111/jvim.16682

**Published:** 2023-04-25

**Authors:** Jane Yu, Craig Ruaux, Christine Griebsch, Lara Boland, Nadia Wong, Peter Bennett, Valerie C. Wasinger

**Affiliations:** ^1^ Sydney School of Veterinary Science, Faculty of Science The University of Sydney Sydney New South Wales 2006 Australia; ^2^ McIvor Road Veterinary Centre Bendigo Victoria Australia; ^3^ Bioanalytical Mass Spectrometry Facility, Mark Wainwright Analytical Centre University of New South Wales Sydney New South Wales Australia

**Keywords:** biomarkers, food‐responsive enteropathy, inflammatory bowel disease, proteins, proteomics

## Abstract

**Background:**

Chronic enteropathy (CE) is common in dogs and can occur with multiple etiologies including food‐responsive enteropathy (FRE) and idiopathic inflammatory bowel disease (IBD).

**Hypothesis/Objective:**

To study the protein profile and pathway differences among dogs with FRE, IBD, and healthy controls using serum proteome analysis.

**Animals:**

Nine CE dogs with signs of gastrointestinal disease and histologically confirmed chronic inflammatory enteropathy and 16 healthy controls.

**Methods:**

A cross‐sectional study with cases recruited from 2 veterinary hospitals between May 2019 and November 2020 was performed. Serum samples were analyzed using mass spectrometry‐based proteomic techniques.

**Results:**

Proteomic profiles showed marked variation in relative protein abundances. Forty‐five proteins were significantly (*P* ≤ .01) differentially expressed among the dogs with CE and controls with ≥2‐fold change in abundance. The fold change of dogs with IBD normalized to controls was more pronounced for the majority of proteins than that seen in the dogs with FRE normalized to control dogs. Proteins involving reactive oxygen species, cytokine activation, acute phase response signaling, and lipid metabolism were altered in dogs with CE.

**Conclusions and Clinical Importance:**

Cytokine alterations, acute phase response signaling, and lipid metabolism are likely involved in pathogenesis of CE. Although there are insufficient current data to justify the use of proteomic biomarkers for assessment of CE in dogs, our study identifies potential candidates.

**Abbreviations**
ACTH: adrenocorticotropic
ARE: antibiotic‐responsive enteropathy
CCECAI: canine chronic enteropathy clinical activity index
CE: chronic enteropathies
cPLI: canine pancreatic lipase immunoreactivity
cTLI: canine trypsin‐like immunoreactivity
FRE: food‐responsive enteropathy
IBD: idiopathic inflammatory bowel disease
IFNγ/IFNG: interferon‐gamma
IL4: interleukin 4
IL6: interleukin 6
MAPK3: mitogen‐activated protein kinase 3; NAD, nicotinamide adenine dinucleotide
NRE: non‐responsive enteropathy
PLE: protein losing enteropathy
PON1: serum paraoxonase and arylesterase 1
PRDM1: PR domain zinc finger protein 1
ROS: reactive oxygen species
SMARCA4 SWI/SNF: related, matrix associated, actin dependent regulator of chromatin, subfamily A member 4
Th: T helper

## 1 INTRODUCTION

Chronic enteropathies (CEs) are common in dogs and are characterized by persistent or recurrent signs of gastrointestinal disease such as vomiting, diarrhea, weight loss, or inappetence for at least 3 weeks.^1^, ^2^ According to treatment responses, CE can be further classified as food‐responsive enteropathy (FRE), antibiotic‐responsive enteropathy (ARE), idiopathic inflammatory bowel disease (IBD), and non‐responsive enteropathy (NRE).^1^, ^2^, ^3^

Diagnosis of the underlying cause of CE can be challenging. Initial assessment revolves around exclusion of extra‐gastrointestinal diseases and other primary gastrointestinal diseases such as infectious causes, parasitism, and neoplasia. Definitive diagnosis of the process leading to CE relies on response to dietary modification, and in dogs failing diet trials, histopathological evaluation of the gastrointestinal tract via surgical or endoscopic biopsies.^1^, ^2^ Yet, in some cases, differences in histological interpretations and interobserver variability among pathologists can be problematic.^1^, ^4^ Furthermore, histopathological findings often do not correlate with treatment response.^3^ An extended amount of time and expense might be spent on treatment trials, and management, leading to owner fatigue and financial exhaustion. In recent years, minimally invasive diagnostic approaches using biomarkers have been explored in diagnosing, monitoring and assessing disease severity in CE.^1^, ^3^, ^5^

Serological biomarkers investigated in dogs with CE include cobalamin, folate, C‐reactive protein, perinuclear antineutrophilic cytoplasmic antibodies, citrulline, soluble receptor for advanced glycation end products and metabolite profiles.^1^, ^3^ Only a few serum biomarkers are commercially available for clinical use. In human gastroenterology, serum protein biomarkers are used to diagnose, monitor treatment response, and to differentiate between different forms of CEs.^6^, ^7^, ^8^, ^9^, ^10^, ^11^ The proteomic profile in dogs for diseases including infectious diseases,^12^, ^13^, ^14^, ^15^, ^16^ neoplasia,^17^ myxomatous mitral valve disease,^18^ heartworm disease,^19^ immune mediated polyarthritis,^20^ chronic hepatitis,^21^ chronic bronchitis, idiopathic pulmonary fibrosis,^22^ meningoencephalitis,^23^ cervical spondylomyelopathy,^24^ chronic kidney disease,^25^ and obesity is reported.^26^ Studies investigating proteomics in gastrointestinal disease remain sparse in the veterinary literature. Given the extensive research and use of proteomics in human gastroenterology, serum proteomics might provide an alternative minimally invasive method for diagnosing, assessing disease severity, monitoring treatment response, and act as a prognostic indicator in dogs with CE. The aim of our study was to identify differences in serum proteins in dogs with CE compared to healthy control dogs, to compare differences in serum proteins between dogs with FRE and IBD, and to further understand the pathophysiology of CE in dogs.

## 2 MATERIALS AND METHODS

### 2.1 Sample collection

Dogs were recruited from 2 veterinary hospitals including a referral hospital (University Veterinary Teaching Hospital, Sydney School of Veterinary Science) and a first opinion clinic (McIvor Road Veterinary Centre) from May 2019 to November 2020. Dogs with persistent or recurrent signs of gastrointestinal disease such as vomiting, diarrhea, weight loss, or a combination of these clinical signs for at least 3 weeks were recruited. The signalment, history, current diet, physical examination findings, diagnostic test results, treatment, and comorbidities of all dogs were retrospectively reviewed. Complete blood count, serum biochemical analyses, and urinalysis were performed in all dogs. Cobalamin concentrations and C‐reactive protein concentrations were also performed in some dogs. As part of the diagnostic investigations for dogs with CE, resting cortisol or adrenocorticotropic (ACTH) stimulation test, canine trypsin‐like immunoreactivity (cTLI), pre‐ and post‐prandial bile acids test or fasting single ammonia test, abdominal ultrasound or computed tomography scan, fecal flotation and PCR analyses, canine pancreatic lipase immunoreactivity (cPLI) or a combination of these diagnostic tests were performed in some dogs at the discretion of the attending veterinarian to exclude extra‐gastrointestinal diseases and infectious causes of gastrointestinal disease. Dogs diagnosed with extra‐gastrointestinal diseases, parasitic and infectious causes of gastrointestinal disease, and dogs without histopathological evidence of gastrointestinal inflammation, were excluded. All dogs had endoscopic biopsies collected. Five dogs had gastroduodenoscopy only, while 4 dogs had both gastroduodenoscopy and colonoscopy performed. Endoscopic evaluation of the gastrointestinal tract was performed based on the World Small Animal Veterinary Association (WSAVA) Gastrointestinal Standardization Group guidelines.^1^, ^4^ The diagnosis of IBD was made based on histopathological evaluation of the biopsied samples. The canine chronic enteropathy clinical activity index (CCECAI) was used to quantify clinical disease activity of all dogs based on recorded history, physical examination findings, and diagnostic test results.^27^

Dogs with CE were classified as FRE, IBD, and NRE based on their treatment response. Dogs that responded to dietary therapy and did not require additional therapeutic trials were defined as FRE. Dogs that did not respond to dietary therapy but responded to immunosuppressant therapy were defined as IBD. Dogs that did not respond to dietary modification, antibiotic therapy, and immunosuppressant treatment were defined as NRE.

Apparently healthy control dogs were recruited from among staff‐ or client‐owned dogs presented for annual health checks and vaccination. They were identified as “healthy” by history and physical examination. An attempt was made to match the cases by age, sex, and body condition score as closely as possible. Blood samples were collected via jugular venipuncture and serum was separated by centrifugation. The serum was then collected into a serum tube and stored at −20°C before analysis.

### 2.2 Protein sample preparation

Total protein concentration was assessed using the 2D Quant kit (Cytiva, Massachusetts, USA) as per manufacturer instructions. Preparation of samples was as described.^28^ Briefly, samples of 100 μg total protein were mixed in 50 μL AMBIC buffer (50 mM Ammoniumbicarbonate, 10 mM DTT, 2 M urea at pH 8) and digested with trypsin at 25°C for 16 hours in a 1:100 enzyme‐to‐protein ratio based on the calculated serum protein concentration. Digestion was halted by acidification. Each sample was then dried and reconstituted in 50 μL of 0.1% formic acid and desalted using C18 stage tips (Thermo Scientific, Illinois, USA) according to the manufacturer's recommendations except that the elution buffer consisted of 80% CH_3_CN, 0.1% formic acid.

### 2.3 Mass spectrometry

Digested peptides were reconstituted in 10 μL 0.1% formic acid and separated by nano‐LC using an Ultimate 3000 HPLC and autosampler (Dionex, Amsterdam, Netherlands) and followed methods similar to those described previously.^28^ Briefly, the sample, 1.7 μL from 10 μL, was loaded onto a micro C18 pre‐column (300 μm × 5 mm, Dionex) with H_2_O:CH_3_CN (98:2, 0.1% TFA) at 10 μL min^−1^. After washing, the pre‐column was switched (Valco 10 port valve, Dionex) into line with a virgin fritless nanocolumn (75 μm i.d × 20 cm) containing reverse phase C18 media (1.9 μm, 120 Å, Dr. Maisch HPLC GmbH). Peptides were eluted using a linear gradient of H_2_O:CH_3_CN (98:2, 0.1% formic acid) to H_2_O:CH_3_CN (64:36, 0.1% formic acid) at 250 nL/min over 120 min. The QExactive (Thermo Electron, Bremen, Germany) mass spectrometer was run in DDA mode where a high voltage of 2000 V was applied to a low volume union and the column (45°C) positioned 0.5 cm from the heated capillary (275°C). A survey scan 350‐1750 m/z was acquired in the Orbitrap (resolution 70 000 at 200 m/z) with an accumulation target of 10^6^ ions, lock mass enabled and up to the 10 most abundant ions (AGC target set to 10^5^, minimum AGC target set to 1.5 × 10^4^) with charge states ≥+2 and ≤+6 sequentially isolated and fragmented.

### 2.4 Protein characterization

Protein dataset‐peak lists were generated from raw files using Mascot Daemon v2.5.1 (Matrix Science, London, UK, www.matrixscience.com). All MS/MS spectra were searched against the SwissProt database (downloaded Jan 2021; 563 972 sequences) for protein identification with the following criteria: allowed 1 missed cleavage; variable modifications oxidation (M), deamidation (R), carbamidomethyl (C), and phosphorylation (S,T,Y); peptide tolerance, ±5 ppm; fragment tolerance, ±0.5 Da; peptide charge +2 to 4+; and enzyme specificity set to semi‐tryptic. A decoy database search was also performed. Scaffold Software (version 4.6.1, Proteome Software Inc., Portland, Oregon, USA) was used to record the protein profiles using spectral counting and was controlled by the Benjamini‐Hochberg procedure for multiple comparisons. An adjusted false discovery rate (FDR), with significance set to *P* < .05 and at least 2 peptides per protein identifications were accepted as valid.

For bioinformatics analysis, Protein ANalysis THrough Evolutionary Relationships (PANTHER) was used to investigate molecular function, biological processes, and protein classes.^29^ Only differentially expressed proteins between dogs with CE and controls with fold change ≥2 were included in this analysis.

Causal networks were also analyzed with Ingenuity Pathway Analysis (Qiagen) to show the interactions between proteins and physical pathways. Graphical networks for these proteins were constructed based on their connectivity algorithms.

### 2.5 Statistical analysis

Descriptive statistics of dog information including age, weight, CCECAI, cobalamin concentrations, and C‐reactive protein levels were generated using R software.

The top protein candidates are identified using filters based on group means, statistically significant at *P* ≤ .01, significance >2‐fold change, FDR, and 95% confidence intervals. Significant changes in expression of protein fold change were analyzed using Fisher's exact test. Fold change ratios were calculated for dogs with CE, FRE and IRE, and normalized to control abundances.

## 3 RESULTS

### 3.1 Dog characteristics

Sixty‐two dogs were initially included: 31 dogs with chronic signs of gastrointestinal disease and 31 control dogs. After exclusion of dogs with extra‐gastrointestinal diseases, comorbidities, and dogs without intestinal biopsies, a total of 25 dogs were included in the analysis. Nine dogs with a histological diagnosis of inflammatory CE were used. All dogs with CE had endoscopic biopsies to confirm the presence of gastrointestinal inflammation. Gastric and duodenal biopsy samples were available from all dogs with CE. Four dogs had colonic samples and 2 dogs had ileal samples. Four dogs had lymphoplasmacytic enteritis, 1 dog had eosinophilic enteritis and 4 dogs had mixed lymphoplasmacytic and eosinophilic enteritis. The median age of CE dogs was 5.0 years (range 3.0‐9.0 years). There were 3 intact males, 4 neutered males, and 2 neutered females. The body condition score ranged from 3 to 6 out of 9, with a median score of 4. Breeds included Australian Bulldog,^1^ Cavoodle,^1^ German Shepherd cross,^1^ Great Dane,^1^ Miniature Pinscher,^1^ Miniature Schnauzer,^1^ Shetland Sheepdog,^1^ Shih Tzu cross,^1^ and West Highland White Terrier.^1^ Three dogs were classified as FRE, 5 dogs had IBD, and 1 dog had NRE. Two dogs had comorbid protein losing enteropathy (PLE). One dog with PLE had lymphoplasmacytic enteritis and another dog had mixed lymphoplasmacytic and eosinophilic enteritis, both dogs were responsive to immune suppression. The dog with NRE did not respond to 2 dietary trials including a hydrolyzed protein diet, metronidazole, prednisolone or cyclosporine, and died of unknown cause. All but 1 dog with CE had been on dietary trials with hydrolyzed, novel protein or antigen‐restricted diets previously. Diet recorded at the time of sample collection included prescription hydrolyzed protein diets, novel protein diets, and an antigen‐restricted diet of crocodile. Three out of 5 of the dogs with IBD had at least had 2 different dietary trials including a prescription hydrolyzed protein diet before immunosuppressants were commenced. Another dog with IBD was administered prednisolone by the referring veterinarian after 1 dietary trial with a prescription hydrolyzed protein diet. Both dogs with PLE were not on dietary trials nor low fat diets at the time of sample collection. The dog that did not undergo a dietary trial before immunosuppressant therapy had severe disease with a CCECAI score of 8 and PLE. The dog was treated with prednisolone 1 day after endoscopic biopsies were obtained because of concerns for rapid deterioration and inappetence. A dietary trial with a novel protein diet was commenced 3 weeks later when the dog's appetite returned to normal. This dog was classified as having IBD. The other dog with PLE had failed 2 dietary trials including a prescription hydrolyzed protein diet, metronidazole therapy, and had poor response to prednisolone treatment at referral. After endoscopy, the dog was then treated with chlorambucil while prednisolone was continued and the dog had a good response. Seven dogs with CE had been treated with metronidazole at a dosage of 10‐23 mg/kg q12h with minimal responses, thus none of the studied dogs were categorized as ARE.

Immunosuppressant therapy used included prednisolone (n = 6), cyclosporine (n = 3) and chlorambucil (n = 1). Prednisolone was administered at an initial dosage of 1.2‐4.4 mg/kg q24h or in divided doses. Cyclosporine was administered at an initial dosage of 6‐8.8 mg/kg once daily or in divided doses and chlorambucil was administered at an initial dosage of 0.1 mg/kg q24h. Immunosuppressants were not used or tapered at consistent doses or frequencies. Four dogs, including 1 dog with PLE, were being treated with prednisolone at the time of sample collection. The CCECAI score ranged from 4 to 16, with a median of 7.

Complete blood count, serum biochemical analyses, urinalysis, and abdominal ultrasound were performed in all dogs with CE. The median C‐reactive protein, serum cobalamin, resting or post ACTH cortisol and pre‐prandial bile acids concentrations are shown in Table 1. Two dogs with CE had abnormally high C‐reactive protein concentrations (RI < 10.7 mg/L). Serum cobalamin concentrations were determined in all but 1 of the dogs with CE before cobalamin supplementation. One dog did not have initial cobalamin concentration determined because cobalamin supplementation had been commenced by the referring veterinarian before referral. Two dogs had cobalamin concentrations below this reference interval. Resting cortisol or ACTH stimulation test was available in all but 1 dog with CE, this dog was receiving prednisolone at the time of referral (Table 1). All dogs with CE were up to date with anthelmintics. Fecal analysis was available for all but 1 dog with CE. Fecal flotation was negative in all tested dogs. Six dogs were positive for *Clostridium perfringens* with low range of *Clostridium perfringens* enterotoxin A gene detected. Liver function tests (pre‐ and/or post‐prandial bile acids test or fasting single ammonia) were available for 4/9 dogs. The post‐prandial bile acids in 1 dog was 6.28 mg/mL (RI: 0‐8.24 mg/mL). Ammonia level of 1 dog was 0.0852 mg/L (RI: 0‐1.67 mg/L). cTLI for 4 dogs ranged from 21 to >50 ug/L (RI: 5.4‐32 ug/L). Snap cPLI results on 2/9 dogs were normal. The urine protein: creatinine ratios of both dogs with PLE were not consistent with protein losing nephropathy.

**TABLE 1:** Characteristics of 9 dogs with chronic enteropathy (CE) and 16 healthy controls by sex, age, body condition score and canine chronic enteropathy clinical activity index (CCECAI), cobalamin, C‐reactive protein, resting cortisol concentrations or adrenocorticotropic (ACTH) stimulation test results, and pre‐prandial bile acids concentrations.

Characteristic	CE dogs (n = 9)	Controls (n = 16)
Sex, n (%)		
Male	7 (78)	6 (38)
Female	2 (22)	10 (63)
Age (years), median (range)	5.0 (3.0‐9.0)	6.0 (2.0‐13.0)
Body condition score, median (range)	4 (3‐6)	5 (4‐6)
CCECAI, median (range)	7 (4‐16)	…
Cobalamin (ng/L), median (range)^a^	436 (<150‐877)	(RI: 203‐1382)
C‐reactive protein (mg/L), median (range)^b^	7.4 (0.1‐20.9)	(RI: <10.7)
Resting cortisol (ug/dL), median (range)^c^	3.40 (2.14‐9.28)	(RI: 0.543‐6.16)
ACTH stimulation test^c^ Baseline cortisol (ug/dL), median (range)	1.3 (<1.01‐5.51)	…
Post ACTH cortisol (ug/dL), median (range)	11.5 (9.17 to 12.1)	…
Pre‐prandial bile acids mg/mL, median (range)^d^	1.96 (0.785‐2.36)	(RI: 0‐4.32)

Abbreviation: RI, reference intervals.

^a^ Cobalamin available in 8 dogs.

^b^ C‐reactive protein available in 6 dogs.

^c^ Resting cortisol available in 5 dogs. ACTH stimulation test available in 3 dogs.

^d^ Pre‐prandial bile acids available in 3 dogs.

Fifteen control dogs were excluded because of comorbidities after reviewing dog records. Sixteen clinically healthy dogs were included as controls in the study including 6 neutered males and 10 neutered females. The median age was 6.0 years (range 2.0‐13.0 years). Breeds included Cross breed,^7^ Staffordshire Bull Terrier,^2^ Belgian Shepherd,^1^ Border Collie,^1^ Chihuahua,^1^ Doberman,^1^ Greyhound,^1^ Jack Russell Terrier,^1^ and Labrador Retriever.^1^ The demographic characteristics of 9 dogs with CE and 16 healthy controls are shown in Table 1. There were no significant differences in age (*P* = .98), sex (*P* = .05) and body condition score (*P* = .12) between dogs with CE and clinically healthy dogs.

The median protein concentrations of samples in CE and controls measured using the 2D Quant kit were 109 mg/mL (range 53.9‐138 mg/mL) and 95.8 mg/mL (range 51.4‐145 mg/mL) respectively. There were no significant differences in median protein concentrations between both groups (*P* = .52).

### 3.2 Changes in the serum protein profile

The total number of proteins identified across all categories was 685, with 578 proteins identified in the CE group and 648 proteins identified in the control group. A total of 208 proteins were detected in common between dogs with CE and controls. Differences were detected between dogs with CE as a whole and the control group, with further differences in relative abundance observed between dogs with IBD and FRE. Only proteins of importance, identified by at least 2 peptides, were further evaluated. Forty‐five proteins were differentially abundant between dogs with CE and controls with fold change ≥|2| (*P* ≤ .01). Thirty‐five proteins were upregulated while 10 proteins were downregulated. The top 13 candidates with greatest fold change ≥|5| in dogs with CE compared to controls (*P* < .01) are shown in Table 2. The fold change ratios of dogs with FRE and IBD compared to controls are also shown in Table 2. The fold change in dogs with IBD normalized to controls was more pronounced for the majority of proteins of interest than that seen in the FRE normalized to controls. Potential candidate proteins with differential abundance were identified based on SD, mean differences, and power calculation (power > 95%) and are illustrated in Table 2. There was no significant difference in protein abundance between dogs that were receiving prednisolone (n = 3) and dogs that were not treated with prednisolone (n = 6) during sample collection (*P* > .01).

**TABLE 2:** Significantly modulated proteins related to enteropathy in dogs with greatest fold change ≥ |5| in dogs with chronic enteropathy (CE) normalized to controls (*P* < .01), fold change in dogs with food‐responsive enteropathy (FRE) and dogs with idiopathic inflammatory bowel disease (IBD) normalized to controls.

Identified proteins	Accession number	Fisher's exact test (*P* value) for CE/control	Fold changes CE	Fold change FRE	Fold change IBD	Potential candidates CE/control	Potential candidates FRE/IBD
Transferrin receptor protein 1	Q9GLD3	<.001	37.3	22.7	43.2	X	X
Gelsolin	Q3SX14	<.001	14.8	9.67	17.2	X	X
Inter‐alpha‐trypsin inhibitor heavy chain H2	Q61703	<.001	14.2	10.7	16.3	X	…
Lumican (fragment)	O46379	<.001	13.3	16.0	14.4	X	…
Alpha‐1‐macroglobulin	Q63041	<.001	8.89	8.53	10.9	X	…
Actin, cytoplasmic 2	P63257	<.001	8.38	6.86	11.0	X	…
Alpha‐1‐antitrypsin	P50447	<.001	6.56	4.33	8.20	X	…
Apolipoprotein C‐I	P56595	<.001	6.13	5.60	7.20	X	…
Fibronectin	P07589	<.001	5.68	3.18	6.24	X	X
Complement C3	P01025	<.001	5.38	4.53	5.73	X	…
Beta‐2‐glycoprotein 1	P33703	<.001	−17.4	−17.4	−14.5	X	…
Serotransferrin	P27425	<.001	−8.24	−5.17	−12.2	X	…
Retinol‐binding protein 4	P18902	<.001	−7.13	−7.13	−7.13	…	…

43  0  −18

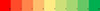

*Note*: Red color indicates the highest upregulated proteins and green color indicates downregulated proteins. Other colors (orange, yellow) indicate intermediate fold changes. The differential ability between 2 groups are illustrated based on SD, mean differences and power >95%, potential candidates as protein biomarkers are marked with “X.”

### 3.3 Gene ontology analysis

**FIGURE 1:**
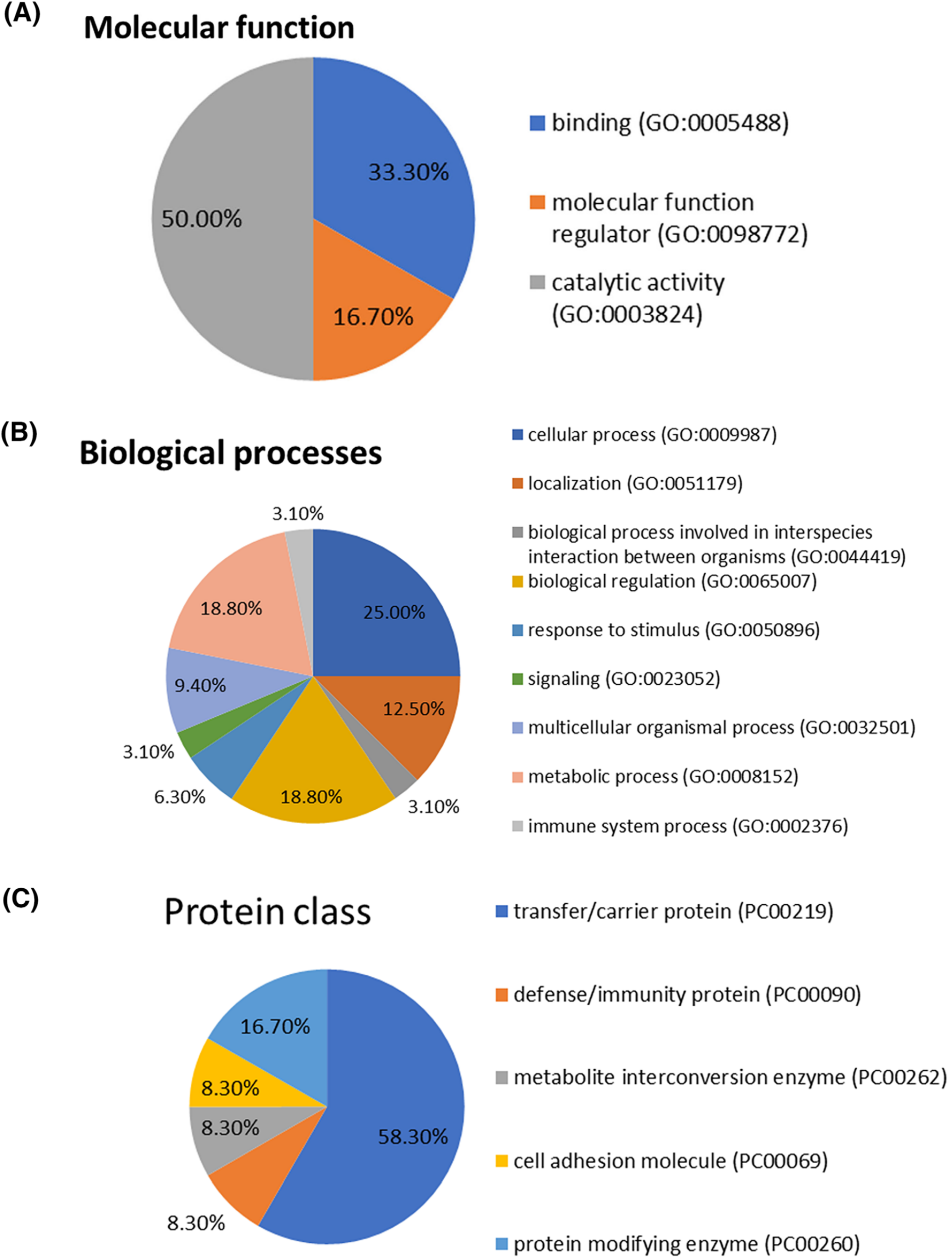
(A) Molecular functions of ≥2‐fold increased protein in abundance and significance in dogs with chronic enteropathy (CE) compared to controls. (B) Biological processes of ≥2‐fold increased protein in abundance and significance in dogs with CE compared to controls. (C) Protein classes of ≥2‐fold increased protein in abundance and significance in dogs with CE compared to controls.

Proteins that were differentially abundant between dogs with CE and controls with fold change ≥2 were used for gene ontology analysis and were categorized according to molecular function, biological processes, and protein classes (Figure 1A‐C). Molecular function characterizes proteins according to molecular level functions while biological process determines the broader function that can be carried out by multiple molecular activities. Half of the modulated proteins in CE have a catalytic function, with a third of proteins performing a ligand or receptor role (Figure 1A). One quarter of proteins participated in cell maintenance processes, with over 18% involved in regulation such as control of gene expression or metabolic processes (Figure 1B). The identified important protein classes reflect gene ontology findings with most modulated proteins (58.3%) found to be transfer or carrier proteins while 16.7% of proteins were protein modifying enzymes. The remaining proteins were involved in immunity, metabolite interconversion enzymes or cell adhesion (Figure 1C).

### 3.4 Lipid metabolism and cytokine activation of IFNγ in dogs with CE

**FIGURE 2:**
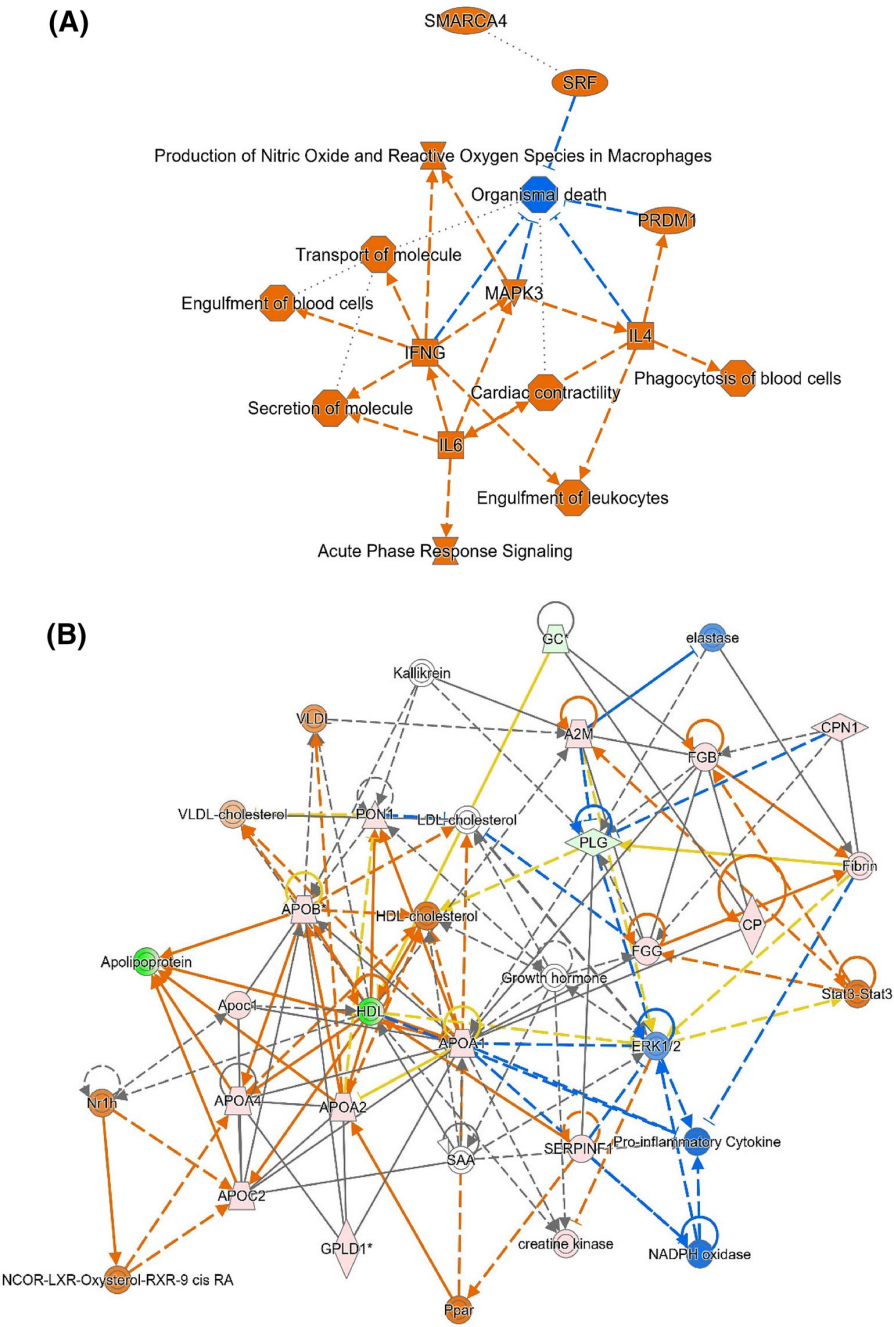
(A) Summary figure of the enriched canonical pathways. Activated pathways are illustrated by orange color, inhibited pathways are illustrated by blue color. Cytokine IL6 activates release of IFNγ (labeled as IFNG on diagram) is a key instigator of inflammatory processes. IFNγ also activates MAPK3 leading to further activation of nitric oxide and reactive oxygen species (ROS) production. Orange shows increased abundance of identified proteins. (B) The lipid metabolism pathway with protein interactions. Increased apolipoproteins, activation (orange) of peroxisome proliferator activator receptor (Ppar) and cholesterol efflux are related to ROS and inflammation leading to inhibition (blue) of proinflammatory cytokines and NAD energy metabolism. Stat3 also leads to activation (orange) of chains of fibrinogen (FGB, FGG) which in turn activates fibrin and then plasminogen (PLG) and ceruloplasmin (CP). Green shows reduced abundance and orange shows increased abundance of identified proteins. Other colors (pink) indicate intermediate abundance.

Differentially abundant proteins between dogs with CE and controls with ≥2‐fold change were also mapped into enriched pathways with Ingenuity Pathway Analysis. The top canonical pathways identified in dogs with CE include activation of Liver × receptor/Retinoid × receptor/Farnesoid × receptor, acute phase response signaling, atherosclerotic signaling and endocytosis signaling pathways (*P* < .001). With upstream regulators interleukin 6 (IL6) cytokine, Mitogen‐activated protein kinase 3 (MAPK3), and transcriptional activators of lymphocytes including B lymphocytes and natural killer cells are also involved. Lipid metabolism, molecular biochemistry, and protein synthesis were identified as the top networks affected in CE. Other upstream regulators with significance values, *Z*‐activation scores and proteins involved in the pathways are shown in Table 3. In addition to cytokines such as interleukin 4 (IL4), interleukin 6 (IL6) and interferon‐gamma (IFNγ), transcriptional regulator PRDM1, transcription activator SMARCA4, and transcription factor protein serum response factor were also involved in the pathway. The pathway provides various functions including transport and secretion of molecules, immune function with phagocytosis and engulfment of leukocytes and cardiac contractility. A summary figure of the enriched canonical pathways is given in Figure 2A. Significantly enriched canonical pathways are reactive oxygen species (ROS) production (Figure 2A) and acute phase response signaling. The lipid metabolism pathway is shown in Figure 2B.

**TABLE 3:** Upstream regulators with molecule type, *Z*‐activation scores, *P* value, and proteins involved are shown.

Upstream regulator	Molecule type	Activation *z*‐score	*P* value	Target molecules/proteins identified
Serum response factor (SRF)	Transcription regulator	2.4	<.001	A2M, CKM, GSN, TLN1, TTN, VCL
Interleukin 4 (IL4)	Cytokine	2.2	.02	APOE, FGB, FGG, GPLD1, TFRC, TLN1
Mitogen‐activated protein kinase 3 (MAPK3)	Kinase	2.0	<.001	APOA1, APOB, FGB, THBS1
PR domain zinc finger protein 1 (PRDM1)	Transcription regulator	2.0	.002	F5, F9, FGB, FGG
Interferon gamma (IFNγ)	Cytokine	2.3	.01	A2M, C4A/C4B, CFB, CP, FGG, GC, TFRC, THBS1
Interleukin 6 (IL6)	Cytokine	2.2	<.001	A2M, ALB, APOA1, APOB, APOE, CLU, CP, CPS1, FGB, FGG, PLG, PON1, TFRC, THBS1
SWI/SNF Related, Matrix Associated, Actin Dependent Regulator of Chromatin, Subfamily A member 4 (SMARCA4)	Transcription regulator	2.0	<.001	A2M, ALB, APOA1, CKM, CP, CPS1, FGG
Signal transducer and activator of transcription 3 (STAT3)	Transcription regulator	1.9	.002	A2M, APOA4, CFB, FGB, FGG, THBS1
Hypoxia inducible factor 1A (HIF1A)	Transcription regulator	2.0	.02	APOE, TFRC, THBS1, TTN
Fibronectin 1 (FN1)	Enzyme	2.0	<.001	APOE, THBS1, TLN1, VCL
Hepatocyte nuclear factor 4 alpha (HNF4A)	Transcription regulator	1.8	<.001	ALB, APOA1, APOA2, APOA4, APOB, APOC2, APOC3, APOE, APOH, C4A/C4B, CP, F9, FGB, GPLD1, GSN, PLG, PON1, PYGL, TFRC, TLN1
Forkhead box protein O1 (FOXO1)	Transcription regulator	2.0	.01	A2M, ALB, APOC3, THBS1
Hepatocyte nuclear factor 1 homeobox A (HNF1A)	Transcription regulator	2.0	.01	ALB, APOA2, APOB, APOC3, APOH, C8A, C8B, CPN1, FGB, GC, PLG, PROC, TFRC
ATP‐binding cassette transporter (ABCA1)	Transporter	2.0	<.001	APOA1, APOA2, APOB, APOE

Abbreviations: A2M, alpha‐2‐macroglobulin; ALB, albumin; APO, apolipoprotein; C4A/C4B, complement4A/complement4B; C8A, complement C8 alpha chain; C8B, complement C8 beta chain; CFB, complement factor B; CKM, creatine kinase M‐type; CLU, clusterin; CP, ceruloplasmin; CPN1, carboxypeptidase N subunit 1; CPS1, carbamoyl‐phosphate synthase 1; F, coagulation factor; FGB, fibrinogen beta chain; FGG, fibrinogen gamma chain; GC, GC vitamin D binding protein; GPLD1, glycosylphosphatidylinositol specific phospholipase D1; GSN, gelsolin; PLG, plasminogen; PROC, protein C; PYGL, glycogen phosphorylase L; TFRC, transferrin receptor; THBS1, thrombospondin‐1; TLN1, talin1; TTN, titin; VCL, vinculin.

## 4 DISCUSSION

Our study utilized an untargeted and hypothesis‐free mass spectrometry‐based proteomic technique. This investigates differences in serum proteomic profiles of dogs with CEs using mass spectrometry‐based proteomic techniques. The technique allows the large‐scale study of proteins simultaneously and rapid identification of potential protein biomarkers.^30^ Our proteomic study identified significant differences in protein profiles between dogs with CE and healthy controls. Forty‐five proteins were differentially abundant between dogs with CE and controls with fold change ≥ 2. Comparing the fold change ratio of FRE normalized to controls and IBD normalized to controls, the fold changes were greater in the IBD group. This suggests that the changes in protein profile were likely more functionally important in IBD and might indicate that IBD is a more severe disease when compared to FRE. Differentially abundant proteins were mostly involved in transfer or carrier functions (58.3%). Proteins responsible for immunity, ROS, acute phase response, lipid metabolism, and molecular biochemistry were also found to be enriched in our analysis. Few studies have explored protein profiles in dogs with CE.^31^, ^32^, ^33^, ^34^ Serum tryptophan concentrations are significantly lower in dogs with PLE compared to healthy control dogs.^31^ Methionine, proline, serine, and tryptophan concentrations are lower in IBD dogs compared to healthy controls.^32^ Fecal proteomic analysis using a liquid chromatography mass spectrometry technique has been performed in dogs with CEs.^33^, ^34^ Immunoglobulin J‐chain isoform 1, a covalent bonded component of immunoglobulin A, is only present in feces of dogs with food responsive diarrhea but not in healthy controls.^33^ Pancreatitis‐associated proteins and several acute phase proteins are significantly more abundant in feces of dogs with CE when compared to the healthy control group.^34^

MAPK3 is the top regulator for protein pathways. The proteins associated with MAPK3 are apolipoproteins, fibrinogen and thrombospondin‐1. MAPK3 is a group of enzymes (kinases) used for phosphorylation of amino acids in proteins.^35^ It forms part of a signaling cascade to induce inflammation, producing proinflammatory cytokines.^35^ It has been suggested that MAPK3 plays a role in mediating IBD and inflammation in people.^35^ Similarly, in our study, the upregulated MAPK3, together with its involvement with fibrinogen and thrombospondin‐1 are key indicators of the inflammatory process in dogs with CE. Thrombospondin‐1 is an extensively studied protein biomarker used in diagnosis and treatment monitoring in IBD in people, and is a potential protein biomarker in cats with CE.^36^

Our protein analysis illustrated activation of cytokines IL4, IL6, and IFNγ (Table 3) in protein pathways. While some of these relationships might be linked to inflammation involving acute phase proteins,^37^ the interconnection between lipid metabolism, immune system, coagulation cascade, and extracellular matrix in the pathogenesis of chronic inflammatory enteropathy likely plays a role. IFNγ, the key cytokine involved in enriched canonical pathway in our analysis, plays an important role in inflammation and immune responses.^38^ IL6 is suggested to be a regulator of Th17 and T regulator cells balance.^39^, ^40^ In dogs with IBD, abnormal recognition of commensal bacteria as pathogenic could occur secondary to dysbiosis, abnormal mucosal permeability, or mutations in T helper 17 (Th17) response and production of proinflammatory cytokines inducing inflammation.^41^ This inflammatory process is accompanied by changes in cytokines released by activated leukocytes, triggering an acute phase response. The cytokine profile in dogs with IBD is described for biopsied tissues.^42^, ^43^ Dogs with IBD have altered cytokine profiles showing a mixed T helper 1 (Th1) and T helper 2 (Th2) cytokine activation.^42^, ^43^ The involvement in cytokines IL4 (produced from Th2 response) and IFNγ (produced from Th1 response) in our analysis likely represents a mixed Th1 and Th2 response. The activation of IL6 suggests the involvement of Th17 response. Although there are differences in T lymphocytes subsets in dogs with IBD compared to healthy dogs,^44^ no study has investigated markers of T cell exhaustion in dogs with IBD. Our results showed upregulation of PRDM1 which is an important transcriptional regulator for T cell homeostasis and is highly expressed in exhausted T cells in chronic viral infection.^45^ PRDM1 contributes to the pathogenesis of IBD in people.^46^ Overall, upstream regulators of relevance were identified in our study relating to T‐cell exhaustion, energy metabolism and transcriptional control.

Proteins involved with lipid metabolism were identified in our study including upregulated clusters of apolipoprotein A‐I, A‐IV, B‐100, C‐I, and E. In dogs with CE, lipoprotein profiles are characterized by a lower serum cholesterol, low‐density lipoprotein/high‐density lipoprotein, and apolipoprotein A‐I concentrations to healthy control dogs.^47^ The differences in lipoprotein profiles are attributed to decreased dietary intake, malabsorption, and inflammation in CE dogs.^47^ While the difference in abundance in apolipoproteins in CE and control dogs in our study could be related to diets and their fat contents, the difference is substantial particularly in light of the fact that decreased high‐density lipoprotein with increased apolipoproteins feeds into pathways of cholesterol efflux and dyslipidemia.

Many proteins identified in our analysis are involved in the ROS production pathway. Oxidative stress is associated with the pathogenesis and progression of IBD in people and dogs.^48^, ^49^ ROS are produced by intestinal epithelial cells during mucosal inflammation in IBD.^48^ Excessive production of ROS causes damage of cytoskeleton proteins, alterations in tight junctions and increased permeability of intestinal epithelial cells, leading to barrier disruption.^48^ ROS is higher in dogs with IBD compared to healthy dogs, suggesting a role for oxidative stress in the pathogenesis of IBD.^49^ ROS and nitric oxide pathways are closely linked to the increase in apolipoproteins, PON1 and cholesterol efflux in our study, supporting ROS activation as an important contributor to the pathogenesis of CE in dogs.

Among the proteins identified, transferrin receptor protein 1 is found to be the most significant differentially abundant protein between dogs with CE and controls with fold change 37.33 (*P* < .001). Transferrin receptor protein 1 has important hematopoietic functions,^50^ and is involved in iron metabolism and facilitates iron transfer by binding to iron loaded transferrin.^50^ It maintains intestinal iron homeostasis.^50^ Iron metabolism also takes part in immune defense and inflammation.^51^ It is possible that the increased fold change in transferrin receptor protein 1 in dogs with CE in our study is related to disruption of intestinal epithelial iron homeostasis and inflammation.

Gelsolin is the second most significant differentiating protein between dogs with CE and controls in our study with fold change 14.8 (*P* < .001). This protein is proposed to be a potential biomarker for ulcerative colitis and inflammation in people to predict disease severity, treatment efficacy, and clinical outcomes.^52^, ^53^, ^54^ Gelsolin is a multifunctional protein and has an important role in cytoskeletal remodeling including actin filament severing, capping and nucleating activity.^52^, ^53^, ^54^ It correlates with clinical and endoscopic activities and has a higher sensitivity and specificity than C‐reactive protein for diagnosing ulcerative colitis in humans.^52^ In contrast to our finding where gelsolin was higher in diseased dogs, gelsolin has an inverse relationship with clinical and endoscopic activity in patients with ulcerative colitis, its level was significantly lower in ulcerative colitis patients compared to healthy controls.^52^ Higher gelsolin concentrations is found in intestinal smooth muscle cells in people with Crohn's disease, suggesting an ongoing inflammatory process.^55^ In dogs, only limited studies have investigated the utility of gelsolin.^24^, ^56^, ^57^ The finding of increased gelsolin levels in dogs in our study could be related to disruption of intestinal mucosa and inflammation in CE. Gelsolin could be a potential biomarker for inflammatory CE in dogs.

There are several limitations to our study. Cats and dogs are less well studied compared to other mammalian species used in research. This results in a reliance on cross‐species identification using a generalized mammalian database. Although over 60 samples were initially collected, the non‐standardized methods of data collection across different practices, and between clinicians, created challenges. We used only biopsy confirmed inflammatory CE to increase the specificity and reliability in this discovery phase study. Despite the small sample size with 3 dogs with FRE and 5 dogs with IBD, our study is comparable to the sample sizes used in proteomic discovery phase studies in people and gives basic insight into differences between these 2 population groups. In human medicine, the discovery of proteomic biomarkers is a multi‐phase process where the initial phase involves screening for potential protein biomarkers with a small number of samples using mass spectrometry techniques, followed by verification of the markers using alternative techniques and increased number of samples. A validation phase then tracks the successful (single) biomarker candidate(s) within a large sample cohort.^58^, ^59^ The higher numbers of IBD cases in our cohort of dogs might reflect referral of cases that were more challenging to manage and required biopsies. Each case was managed at the discretion of the attending veterinarian and the case management was not standardized. Not all diagnostic tests were performed in each dog, thus liver function tests, cPLI and cTLI were only available for some dogs. One dog with CE did not have a dietary trial before immunosuppressant therapy because of concerns for potential rapid deterioration and inappetence, it is possible that this dog could have been misclassified as IBD.

With the diversity of test groups, our data cannot be extrapolated to other dogs with CE. Therefore, the utility of proteomic markers to diagnose, monitor treatment or prognosticate CE in dogs cannot be justified based on our study at this time. However, the application of proteomics allows screening and discovery of potential biomarkers. The data reported here provide preliminary information about serum proteomics in dogs with CE and discovers potential candidates for future development. Significant difference in protein profiles were identified, with greater fold differences observed in dogs with IBD compared to those with FRE. The interrelationship between inflammation, cytokine activation, ROS production, acute phase response signaling, and lipid metabolism in the pathogenesis of CE in dogs is characterized using these proteomic techniques, deepening our understanding of both systemic and local factors contributing to the development of CE.

## CONFLICT OF INTEREST DECLARATION

Authors declare no conflict of interest.

## OFF‐LABEL ANTIMICROBIAL DECLARATION

Metronidazole was used off‐label. This antibiotic was used at the discretion of the attending and referring veterinarians.

## INSTITUTIONAL ANIMAL CARE AND USE COMMITTEE (IACUC) OR OTHER REGULATORY BODY APPROVAL DECLARATION

Approved by the Animal Ethics Committee, University of Sydney (project number 2019/1548).

## HUMAN ETHICS APPROVAL DECLARATION

Authors declare human ethics approval was not needed for this study.

## Supporting information


**Table S1.** Power calculations of each group are shown comparing the chronic enteropathy (CE), food‐responsive enteropathy, idiopathic inflammatory bowel disease, and control groups. The power is sufficient (>95%) when comparing CE and control groups in all proteins except retinal binding protein.
