# Supplementary material for: Serum proteome of dogs with chronic enteropathy
Source: J Vet Intern Med. 2023 Apr 25;37(3):925–35. doi: 10.1111/jvim.16682 (PMC10229361; doi:10.1111/jvim.16682)
Supplement: Supplementary file 1 — Table S1. Power calculations of each group are shown comparing the chronic enteropathy (CE), food‐responsive enteropathy, idiopathic inflammatory bowel disease, and control groups. The power is sufficient (>95%) when comparing CE and control groups in all proteins except retinal binding protein. [file JVIM-37-925-s001.pdf]

Supplementary Table 1: Power calculations of each group are shown comparing the CE, FRE, IBD and control groups. The power is sufficient (>95%) when comparing CE and control groups in all proteins except retinal binding protein.

	**Standard deviation**				**Absolute difference between means**				**Power %**			
**Protein**	**Control**	**FRE**	**IBD**	**Total CE**	**CE/ control**	**FRE/ control**	**IBD/ control**	**FRE/ IBD**	**CE/ control**	**FRE/ control**	**IBD/ control**	**FRE/ IBD**
Transferrin receptor protein 1	0.970	0.940	3.31	3.68	9.08	5.42	10.6	5.13	100	100	100	100
Gelsolin	0.00	1.70	1.94	4.02	14.8	9.67	17.2	7.53	100	100	100	99.9
Inter-alpha-trypsin inhibitor heavy chain H2	1.83	6.60	6.52	6.43	9.09	6.65	10.5	3.87	100	32.2	84.1	10.3
Lumican (Fragment)	0.48	1.41	2.40	2.05	1.54	1.88	1.68	0.20	100	51.4	24.8	5.13
Alpha-1-macroglobulin	0.85	1.89	2.42	2.35	2.47	2.35	3.09	0.73	100	45.5	65.8	7.34
Actin, cytoplasmic 2	1.69	4.24	9.60	7.72	3.23	2.56	4.36	1.80	100	14.6	13.0	7.89
Alpha-1-antitrypsin	0.00	3.30	6.18	5.31	6.56	4.33	8.20	3.87	100	49.9	67.8	27.7
Apolipoprotein C-I	1.82	3.74	7.29	6.15	6.42	5.75	7.75	2.00	99.1	63.6	49.5	9.60
Fibronectin	9.47	4.50	19.7	21.9	34.8	16.2	39.0	22.7	100	99.9	95.4	100
Complement C3	6.87	5.56	10.8	10.2	38.1	30.7	41.1	10.5	100	100	100	58.3
Beta-2-glycoprotein 1	12.4	1.41	1.60	1.49	16.4	16.4	16.2	0.20	96.2	100	100	5.13
Serotransferrin	15.6	4.03	2.06	3.17	25.8	23.7	26.9	3.27	96.2	100	100	15.8
Retinol-binding protein 4	6.81	0.00	0.00	0.00	7.13	7.13	7.13	0.00	68.2	34.4	48.5	NA

*Summary Statement: Group sample sizes of 9 and 16 achieve 100% power to reject the null hypothesis of equal means when the population mean difference is 9.08 with standard deviations of 3.68 for group 1 and 0.970 for group 2, and with a significance level (alpha) of 0.05 using a two-sided two-sample equal-variance t-test.
